# Is the use of preoperative breast MRI predictive of mastectomy?

**DOI:** 10.1186/1477-7819-11-154

**Published:** 2013-07-12

**Authors:** Brigid K Killelea, Baiba J Grube, Muhammad Rishi, Liane Philpotts, Eliza-Jasmine Tran, Donald R Lannin

**Affiliations:** 1 Department of Surgery, Yale University School of Medicine, New Haven, USA; 2 Department of Radiology, Yale University School of Medicine, New Haven, USA; 3 The Breast Center-Smilow Cancer Hospital at Yale New Haven, Suite A 20 York St, New Haven, CT 06520, USA

## Abstract

**Background:**

Several recent studies have described increasing rates of unilateral and bilateral mastectomy among women with newly diagnosed breast cancer. The use of breast magnetic resonance imaging (MRI) has also risen rapidly, leading to speculation that the high false-positive rate and need for multiple biopsies associated with MRI may contribute to more mastectomies. The objective of this study was to determine whether newly diagnosed patients who underwent preoperative MRI were more likely to undergo mastectomy compared with those who did not have a preoperative MRI.

**Methods:**

A retrospective review was performed of all newly diagnosed patients with breast cancer at our academic breast center from 2004 to 2009.

**Results:**

The proportion of newly diagnosed patients with breast cancer having MRI prior to surgery increased from 6% in 2004 to 73% in 2009. Of 628 patients who underwent diagnostic MRI, 369 (59%) had abnormal results, 257 (41%) had one or more biopsies, and 73 had additional sites of cancer diagnosed. Patients with a malignant biopsy, or those with an abnormal MRI who did not undergo biopsy, had an increased mastectomy rate (*P*<0.01). However, patients with a normal MRI or a benign biopsy actually had a decreased mastectomy rate (*P*<0.05). Although there was a trend toward more bilateral mastectomies, the overall mastectomy rate did not change over this time period.

**Conclusions:**

Although there is a strong relationship between the result of an MRI and the choice of surgery, the overall effect is not always to increase the mastectomy rate. Some patients who were initially considering mastectomy chose lumpectomy after an MRI.

## Background

Carefully conducted randomized controlled trials have shown no significant difference in either overall survival or local recurrence for women who undergo mastectomy versus those who elect to have breast conservation surgery (BCT) followed by radiation therapy for the treatment of early-stage breast cancer [1]. Based on these findings, mastectomy rates had been declining over the past 3 decades in favor of BCT [2], but within the past several years both unilateral and bilateral mastectomy rates have begun to rise [3,4]. Several reasons for this observed increase have been proposed, including expanded screening for carriers of *BRCA1* and *BRCA2* genetic mutations, advances in post-mastectomy breast reconstruction, increased public awareness and heightened patient anxiety, and the more liberal use of preoperative breast magnetic resonance imaging (MRI). However, there are few data with which to evaluate the significance of these proposed etiologies.

Currently, breast MRI is often used in newly diagnosed patients with breast cancer to evaluate the extent of disease and look for additional foci of mammographically occult lesions in the affected breast, and to examine the contralateral breast. However, because MRI is a very sensitive test, the false-positive rate for detected lesions is high, leading to an increased number of image-guided biopsies before definitive surgery. Nonetheless, MRI does detect mammographically occult disease in the ipsilateral breast in 11 to 31%, of patients [5] and in the contralateral breast in 3 to 4% [6]. Although these lesions are usually relatively small when they are detected, surgical treatment planning can be affected. Based on the extent of disease and the location in the breast, patients may undergo wider lumpectomy or mastectomy; however, for the majority of patients in whom no additional lesions are found, management remains unchanged. Whether or not this increased rate of detection of small foci of disease will ultimately lead to improvements in the local recurrence rate and/or mortality rate remains to be determined.

The purpose of this study was to characterize the increased use of preoperative breast MRI at our institution over a 6 year period, and to investigate whether patients who underwent a preoperative breast MRI were more likely to ultimately have a mastectomy than those who did not undergo MRI.

## Methods

Using a prospectively maintained database, all newly diagnosed patients with breast cancer who were treated at the Yale New Haven Breast Center from 2004 to 2009 were identified. Patients who did not receive definitive breast surgery during the study period because of distant metastases or neoadjuvant chemotherapy were excluded. Patient and tumor characteristics, imaging and biopsy findings, surgical treatment, and final pathology results were recorded. Yearly mastectomy rates were determined and compared with BCT rates across the 6 year period.

This was a retrospective chart review and was approved by the Yale University IRB as exempt.

Patients who underwent preoperative breast MRI were identified. MRI was performed at the discretion of the treating breast surgeon. Those with additional suspicious lesions were identified, and biopsy results were obtained. Operative, imaging, and pathology reports were reviewed and compared with data in the clinical chart for accuracy.

### Statistical analysis

Statistical analysis was performed using SPSS statistical software (SPSS Inc., Chicago, IL, USA). Categorical data were compared with χ^2^ tests and means of continuous data were compared with *t*-tests. Multivariable analysis was performed with logistic regression. All significance testing was two-sided.

## Results

In total, 1,445 patients with newly diagnosed breast cancer had definitive breast surgery at the Yale New Haven Breast Center from 2004 to 2009. Of this group, 628 patients (43%) underwent diagnostic MRI. Patient and tumor characteristics for the two groups are presented in Table 1. The mean age for those patients who underwent breast MRI was 53 years and the mean age for those who did not was 60 years (*P*<0.001) There was no significant difference in ethnicity, tumor stage, tumor histology or mean tumor size between the two groups.

**Table 1:** Patient and tumor characteristics

	**MRI (n = 628)**	**No MRI (n = 817)**	***P*-value**
Age, years			
Mean	53	60	<0.001
<50	43%	26%	<0.001
>50	56%	73%	<0.001
Ethnicity			
Asian	3%	2%	NS
Black	8%	12%	NS
Hispanic	6%	4%	NS
White	78%	79%	NS
Other/unknown	5%	3%	NS
Stage			
0	23%	27%	0.08
1	39%	39%	NS
2	29%	22%	NS
3	7%	8%	NS
4	2%	3%	NS
Tumor size (cm)	1.6	1.5	NS
Histology			
DCIS	24%	26%	NS
Infiltrating ductal	59%	53%	NS
Infiltrating lobular	12%	11%	NS
Other	5%	10%	NS

Abbreviations: *DCIS* ductal carcinoma *in situ*, *MRI* magnetic resonance imaging, *NS* non-significant.

**Figure 1:**
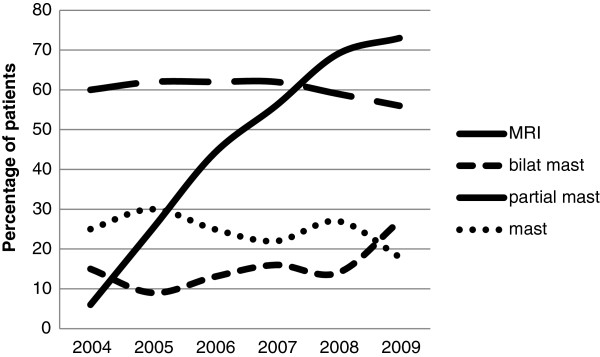
Magnetic resonance imaging (MRI) use and definitive surgery over time.

The use of preoperative MRI increased sharply over the study period, from 6% of newly diagnosed patients with breast cancer in 2004 to 73% in 2009. The rates of lumpectomy and unilateral mastectomy declined slightly over this period, whereas the rate of bilateral mastectomy increased (Figure 1).

The relationship between the type of breast surgery performed and MRI usage is shown in Table 2. The number of partial mastectomies performed over the study period was 355 (57%) in the group who underwent preoperative MRI and 504 (62%) in the group who did not (*P* = 0.06). Thus MRI was associated with a non-significant trend toward a lower lumpectomy rate.

**Table 2:** Surgical treatment for those who had definitive surgery

**Type of surgery**	**MRI (n = 628)**	**No MRI (n = 817)**	***P*-value**
Partialmastectomy	355 (57%)	504 (62%)	0.06
Unilateralmastectomy	147 (23%)	211 (26%)	NS
Bilateralmastectomy	123 (20%)	102 (12%)	<0.005

Abbreviations: *MRI* magnetic resonance imaging, *NS* non-significant.

There was no significant difference between the two groups with regard to unilateral mastectomy (23% and 26%, respectively), but the number of bilateral mastectomies performed was significantly higher in the group that underwent preoperative MRI (123 (20%) versus 102 (12%), *P*<0.005).

Table 3 shows some of the outcomes associated with the use of MRI. Of 628 patients who underwent preoperative breast MRI, 369 (59%) had abnormal results: 29% on the ipsilateral side, 12% on the contralateral side, and 18% in both breasts. In total, 257 patients (41%) underwent one or more biopsies. Most often, targeted ultrasound with image-guided core biopsy was attempted before MRI guided biopsy. Malignant results were documented by biopsy results in the ipsilateral breast in 52 cases (8%), the contralateral breast in 15 (2%), and both in 6 (1%).

**Table 3:** Surgical treatment according to the results of magnetic resonance imaging (MRI)

**MRI results**	**n**	**Lumpectomy**	**Ipsilateral mastectomy**	**Bilateral mastectomy**
No MRI	817	62%	26%	12%
MRI				
Normal	259	66%	17%^c^	16%
Abnormal				
Ipsilateral	182	51%^c^	34%^c^	15%
Contralateral	73	53%	21%	26%^c^
Both	114	46%^c^	23%	31%^c^
MRI biopsy^a^				
None	132	39%^c^	35%^c^	26%^c^
Benign	184	66%	21%	13%
Malignant				
Ipsilateral^b^	52	35%^c^	38%^c^	27%^c^
Contralateral	15	33%^c^	0^c^	67%^c^
Both	6	0^c^	0	100%^c^

^a^For those with abnormal MRI.

^b^In addition to index lesion.

^c^*P*<0.05 compared with the rate for the same surgery with no MRI.

The outcome of the MRI strongly influenced the choice of surgery compared with the group that did not undergo MRI. As might be expected, a diagnosis of a second ipsilateral or contralateral malignancy was associated with a decreased lumpectomy rate and an increase in the rates of unilateral and bilateral mastectomy. However, the group that had an abnormal MRI and did not undergo biopsy also had a lumpectomy rate of only 39%, and the rates of unilateral and bilateral mastectomy were 35% and 26%, respectively. We were not able to determine how many of these women chose mastectomy to avoid another needle biopsy. Although not significant, when compared with the women who did not have an MRI, the women with a normal MRI or a benign biopsy actually had an increased lumpectomy rate (66% and 62%). Thus, some women who were considering mastectomy may have chosen lumpectomy based on the MRI results. This may explain why the use of MRI had a relatively modest effect overall on the lumpectomy rate.

Because young women were more likely to receive an MRI and also more likely to choose bilateral mastectomy, a multivariable logistic regression model was constructed with age, stage, and MRI use. As seen in Table 4, both young age and MRI use were independently associated with an increased rate of bilateral mastectomy.

**Table 4:** Multivariable logistic regression for bilateral mastectomy

**Characteristic**	**OR (95% CI)**	**P value**
Age		
Over 50	Reference	
Under 50	3.47 (2.57 to 4.70)	< 0.001
MRI use		
No	Reference	
Yes	1.38 (1.02 to 1.87)	0.036

Abbreviations: *CI* confidence interval, *MRI* magnetic resonance imaging, *OR* odds ratio.

## Discussion

The use of preoperative MRI in patients with newly diagnosed breast cancer is controversial. Proponents of the test are supported by studies showing that breast MRI detects mammographically occult disease in both the ipsilateral and the contralateral breast, has overall sensitivity rates ranging from 73 to 94%, and has a positive predictive value ranging anywhere from 24 to 89% [7-11]. There is speculation that better preoperative imaging with MRI may lead to decreased numbers of patients with positive margins, and thus lower recurrence rates and ultimately improved overall survival, although to date these data are conflicting [12,13]. Because of the low recurrence rate after BCT, studies designed to show a difference in these outcome measures would require large numbers to achieve statistical significance and long term follow-up to detect recurrence rates, and may be difficult to perform.

On the other hand, preoperative breast MRI has been criticized for several reasons. These include but are not limited to: delays in surgical management, an unacceptably high false-positive rate, increased costs associated with the test itself and with additional biopsies, heightened patient anxiety, and unnecessary changes in surgical management to avoid additional biopsy and/or the perceived risk of a future recurrence. Although individual studies vary, a recent review by Houssami and Hayes reported that, based on pooled estimates from meta-analyses, preoperative breast MRI led to more extensive surgery in 11.3% of patients, including wider resection or mastectomy, and that 8.1% of all women eligible for BCT were treated with mastectomy because of MRI-only detection of additional disease [14].

Nonetheless, the use of preoperative breast MRI has gained momentum in the surgical community. Although its use varies by geographic region, up to 74% of all breast imaging centers offer the test [15]. Thus, it appears that the selective use of preoperative breast MRI is increasingly being used to guide clinical decision-making for newly diagnosed patients. Our data support this trend. Over the study period, there was an increase in both the percentage of newly diagnosed patients who underwent preoperative MRI and the percentage of patients who underwent biopsy for MRI-detected lesions. Although we may still be on the steep portion of the learning curve with regard to the characterization of suspicious lesions seen on MRI, addressing the low specificity of MRI is certainly an area for which further research is warranted. In addition, whether these additional areas might ever become clinically significant, especially among older patients and patients undergoing post-operative radiation therapy, remains an area of debate.

The observed increase in the utilization of preoperative breast MRI and a concurrent rise in the number of unilateral and bilateral mastectomies performed over the past several years have led researchers to wonder if the two events are related. Several recent studies have reported increasing rates of contralateral prophylactic mastectomy, especially among younger, highly educated patients, and those with a positive family history [4,16-18]. Although this phenomenon does coincide with the adoption of preoperative breast MRI, it is not yet clear whether the relationship is one of cause and effect. In the present study, BCT rates did decrease slightly over the study period in favor of mastectomy, going from 60% in 2004 to 54% in 2009, but the use of preoperative MRI increased much more sharply. Interestingly, the highest BCT rates of any group were among patients with a normal MRI, (66%), even compared with those who did not undergo MRI. Perhaps an MRI scan that does not show any additional suspicious findings can help guide patients who are interested in pursuing BCT.

In a recent editorial, Tuttle asked whether patients with abnormal MRI findings and biopsy on the contralateral side might be more inclined to undergo bilateral mastectomy than those who did not [19]. In our study, we examined this question, and found that this was indeed the case. The greatest difference with regard to definitive surgical management was in the type of mastectomy performed that is, unilateral versus bilateral. In 2004, 15% (n = 20) of patients underwent bilateral mastectomy, but by 2009, 27% of patients (n = 56) underwent bilateral mastectomy. During the same period, the use of preoperative MRI increased from 6% to 73%. Compared with other treatment options, those who underwent bilateral mastectomy were more likely to have had a contralateral biopsy-proven malignancy based on MRI findings. Although abnormal breast MRI findings may have influenced the decision to undergo bilateral mastectomy, it is probable that there are a host of factors that affect the choice of definitive surgery for any given patient. It is likely that physician recommendations, patient age, family history, genetic testing, and reconstruction options all play a role.

## Conclusion

In summary, we found a dramatic increase in the use of preoperative breast MRI among newly diagnosed patients with breast cancer over the 6 year period of the study. A preoperative MRI that revealed only the known cancer, or that led to a benign biopsy, was associated with a higher rate of BCT over mastectomy. Thus, although there is a strong relationship between the result of the MRI and the choice of surgery, the overall effect is not always to increase the mastectomy rate.

## Competing interests

The authors declare that they have no competing interests.

## Authors’ contributions

BK designed the research project conception, analyzed the data, wrote the manuscript, and contributed to the development of overall research plan. DL conducted the data analysis, wrote the manuscript, and was responsible for study oversight. MR conducted research (data collection). BG, JT, and LP aided in manuscript writing and editing and contributed to the research plan. BK and DL had primary responsibility for final content. All authors read and approved the final manuscript.
